# A Link Between Dietary Modifications Focused on Unsaturated Fatty Acids and the Course of Oral Autoimmune Diseases: A Systematic Review

**DOI:** 10.3390/jcm15145542

**Published:** 2026-07-15

**Authors:** Aleksandra Diedul, Marta Sikora, Małgorzata Kaniecka, Marzena Liliana Wyganowska, Johan Peter Woelber, Zuzanna Slebioda

**Affiliations:** 1 Medical Faculty, Poznan University of Medical Sciences, ul. Fredry 10, 61-701 Poznań, Poland; 2 Department of Periodontology and Oral Mucosa Diseases, Poznan University of Medical Sciences, ul. Bukowska 70, 60-812 Poznań, Poland; 3 Policlinic of Operative Dentistry, Periodontology, and Pediatric Dentistry, Medical Faculty Carl Gustav Carus, Technische Universität Dresden, 01-307 Dresden, Germany

**Keywords:** unsaturated fatty acids, oral lichen planus, recurrent aphthous stomatitis, pemphigus, autoimmune diseases

## Abstract

**Background/Objectives:** The aim of this systematic review was to analyze the impact of dietary modifications on unsaturated fatty acids (UFAs) intake on the course of three oral autoimmune conditions: oral lichen planus (OLP), recurrent aphthous stomatitis (RAS), and pemphigus. These acids serve a structural function as key components of cell membrane phospholipids, influencing their fluidity and membrane receptor activity. The proper ratio of these acids in the diet may be crucial for maintaining normal functioning of the human immune system. **Methods:** The following bases were searched with no time restrictions: PUMS Library, Embase/Elsevier, PubMed/MEDLINE, Scopus, and Web of Science. Of the 247 results identified in pre-screening, 6 studies that met all search criteria were included in the final systematic review. The risk of bias was assessed using the Revised Cochrane Risk of Bias Tool for randomized trials and the Newcastle–Ottawa Scale for non-randomized studies. **Results:** The outcome measures included: proportions of LA-derived oxylipins (9-HODE, 13-HODE), ulcer size (mm), monthly number of ulcers, average pain (VAS), duration of episodes, and quality of life (OHIP-14), healing time, and fatty acid analysis of total plasma phospholipid fraction, Pemphigus Disease Area Index (PDAI) for disease severity (mild ≤ 15, severe > 15); AHEI-2010 score (9–90 points) for diet quality. In all studies, improvements in clinical parameters and subjective complaints were observed after implementing a dietary intervention. **Conclusions:** Dietary modification may be a valuable component of adjunctive therapy. Nutritional optimization offers a genuine opportunity to reduce reliance on conventional pharmaceuticals, thereby limiting the risk of systemic adverse effects associated with long-term pharmacotherapy.

## 1. Introduction

A steady rise in the frequency of autoimmune diseases in the last few decades has been observed. Oral manifestations of autoimmune disease are frequently the primary signs of those conditions, but sometimes the oral region may be the only involved site. Contemporary diagnostics of oral mucosal diseases with an autoimmune etiology encompasses a broad spectrum of conditions, including oral lichen planus (OLP), recurrent aphthous stomatitis (RAS), and pemphigus [1,2,3,4]. These conditions are characterized by a considerable variability in population prevalence, yet they share a common denominator: immune system autoaggression directed against the body’s own structures [1,2,3,4,5,6]. Disruption of mucosal barrier integrity is observed, resulting from basement membrane destruction in OLP [7], focal epithelial barrier disruption in RAS [8], and acantholysis in pemphigus [2,4]. These processes increase tissue permeability to external factors, thereby amplifying the local immune response. Despite differences in underlying immunopathological mechanisms of these diseases, they share a common feature of immune system dysregulation, which is manifested in the oral mucosa. A comparison of epidemiological parameters, immunological mechanisms, and typical clinical manifestations for the discussed disease entities is presented in Table 1.

Currently, available treatment approaches are of limited efficacy. The standard therapeutic approach focuses on topical or systemic immunosuppression, primarily corticosteroids, to reduce inflammation and restore tissue homeostasis [1,5,8]. Due to several, often very serious, complications associated with this treatment strategy, there is an urgent need to develop a safe and effective supportive strategy to enhance clinical outcomes and patient well-being. Although the exact etiology of OLP, RAS, and pemphigus remains unknown, their pathogenesis is driven by a multifactorial synergy of genetic predisposition, lifestyle, and dietary triggers. We specifically link these conditions to ω-3 polyunsaturated fatty acids (PUFAs) because clinical and biochemical evidence indicates that these diseases share inflammatory markers, such as imbalanced lipid derivatives and oxidative pathways, that are fundamentally modulated by ω-3 intake. By addressing these shared lipid-dependent inflammatory mechanisms, our research investigates whether dietary optimization can offer a unified, adjuvant therapeutic pathway for these diverse oral autoimmune conditions. Genetic factors, particularly specific HLA antigen expressions, alter immune tolerance. These distinct HLA profiles include HLA-A3, HLA-B8, HLA-DR1, HLA-DR9 in OLP, HLA-DRB1*0402, HLA-DQB1*0503 in RAS and HLA-B51, HLA-A2, HLA-B12 in pemphigus. This genetic susceptibility is exacerbated by lifestyle factors, such as chronic psychological stress, which elevate systemic cortisol and pro-inflammatory cytokines, thereby disrupting oral tissue homeostasis [10,11,12,13,14,15]. In diseases characterized by overproduction of inflammatory mediators and immunological dysregulation, including OLP, RAS, and pemphigus, there is reason to suspect that dietary modifications, including adequate intake of ω-3 and ω-6 fatty acids, may be significant for the clinical course of these diseases.

Polyunsaturated fatty acids (PUFAs), constituting a group of essential unsaturated fatty acids (EFAs), represent a series of substances that the human body is unable to synthesize independently. They must be supplied exogenously through an appropriate diet. Two major subgroups are distinguished: ω-3 (*n*-3) and ω-6 (*n*-6). The nomenclature of these compounds is based on the position of the first double bond at the third and sixth carbon atoms, respectively, counting from the methyl end of the carbon chain [10,11]. These acids serve a structural function as key components of cell membrane phospholipids, influencing their fluidity and membrane receptor activity. They also serve as important energy sources in the β-oxidation process and as precursors of lipid mediators that regulate the immune response [12,13]. The most common representatives of ω-6 are linoleic acid (LA) and arachidonic acid (AA) [11,14]. In turn, the most important acids from the ω-3 family include α-linolenic acid (ALA) and its metabolic derivatives: eicosapentaenoic acid (EPA) and docosahexaenoic acid (DHA). The main sources of ω-3 in the human diet are marine sources (such as wild-caught salmon and mackerel), certain plant seeds (such as flaxseed and chia), and animal-based products, including meat, eggs, milk, and cheese, particularly when sourced from wild or pasture-raised animals. Also, green leafy vegetables, fruits, nuts, and berries contain a significant amount of ω-3 fatty acids. In the modern Western diet, people ingest more products rich in ω-6 fatty acids, predominantly found in cereal grains, refined vegetable oils (such as sunflower, corn, and soybean oils), commercially farmed livestock, standard supermarket eggs, and aquaculture-raised fish. As a result, the Western diet has drastically altered the ω-acid ratio, often exceeding 15:11 and sometimes reaching 20:1. This could be crucial for maintaining the immune system’s function. Some studies report that excess ω-6 acids in the diet shift the body’s balance, promoting systemic low-grade inflammation and, consequently, a prothrombotic and pro-contractile vascular effect. The optimal *n*-6/*n*-3 ratio has been established as approximately 1:1 to 4:1, where the inflammatory state appears only when needed [e.g., during infection] and is rapidly resolved [16,17,18,19,20,21,22]. Restoration of balance inhibits chronic activation of immune cells. Constant, excessive readiness of the immune system to attack (ratio observed in Western diet) promotes autoimmunity [17,19,23,24]. Table 2 presents the most relevant biological and immunological effects of unsaturated fatty acids.

The aim of this systematic review was to analyze the impact of dietary modifications related to UFAs intake on the course of three oral autoimmune conditions: OLP, RAS, and pemphigus.

## 2. Materials and Methods

The following bases were searched with no time restrictions: PUMS Library, Embase/Elsevier, PubMed/MEDLINE, Scopus, and Web of Science. A detailed strategy is described in Appendix A and Table A1 presents the summary of the search results. The researchers implemented a language restriction when assessing the records, and only full-text English articles were ultimately qualified for further evaluation. Additionally, a manual search of bibliographies and publications identified through database searches in PubMed, Web of Science, and the Cochrane Library was performed to identify potentially eligible references. To identify missing information or data, we attempted to contact the authors of the relevant studies. The grey literature was not searched for this review. The reviewers were not blinded to the authorship of the analyzed studies. Because the PUMS Library provides access to several databases that were also searched individually (including MEDLINE, Embase, Scopus, and Web of Science), duplicate records were expected. After retrieval, all citations were screened collectively and duplicates were identified using publication title, authors, year of publication, DOI, and study characteristics. Records referring to the same study were removed prior to the eligibility assessment, ensuring that each study was included only once in the final review.

The data from each study entered into this analysis were independently extracted by three reviewers and included: year of publication, country of origin, details of participants, including demographic characteristics, type of intervention and comparisons, study design, and outcomes.

Initially, the records were assessed by three independent authors based on the relevance of the title and/or abstract. Studies selected in the initial search were evaluated in their entirety by the same authors. Full-text articles were considered eligible if they reported original human data on the relationship between dietary modification, unsaturated fatty acids, lipid-related biomarkers, or dietary quality and oral autoimmune diseases (oral lichen planus, recurrent aphthous stomatitis, or pemphigus). Eligible study designs included randomized controlled trials, non-randomized interventional studies, observational studies, cross-sectional studies, and mechanistic studies involving human participants. Mechanistic studies were included when they investigated biological pathways or lipid mediators relevant to disease activity and clinical manifestations. Reviews, meta-analyses, conference abstracts, editorials, protocols, animal studies, and in vitro studies were excluded.

Because of the scarcity of studies investigating dietary fatty acid interventions in oral autoimmune diseases, all original human studies providing relevant clinical or biological outcome data were considered eligible, including randomized controlled trials, interventional studies, observational studies, and cross-sectional studies. This approach was adopted to provide a comprehensive overview of the available evidence and to identify potential associations and knowledge gaps within this emerging field.

The primary and secondary outcome measures included: proportions of LA-derived oxylipins, pain reduction, ulcer size and duration, healing time, recurrence rate, quality of life (OHIP-14), fatty acid analysis of total plasma phospholipid fraction, Pemphigus Disease Area Index (PDAI) for disease severity (mild ≤ 15, severe > 15); AHEI-2010 score (9–90 points) for diet quality, lipid index calculations based on dietary fat intake. Any disagreements between researchers were resolved after consultation with the senior authors.

This review complies with the PRISMA guidelines, and the PRISMA Checklist is provided in the Supplementary Materials. This study protocol has been registered in Open Science Framework (OSF) registry (doi:10.17605/OSF.IO/NCTU3).

A quantitative meta-analysis was not performed due to significant heterogeneity across the included studies.

**Figure 1:**
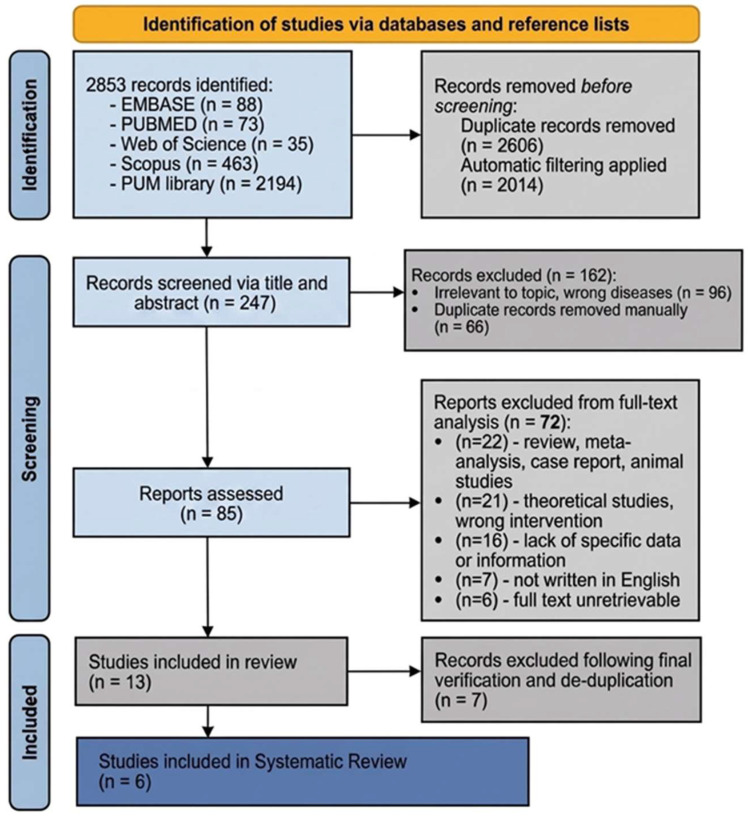
The PRISMA flowchart for this systematic review.

Figure 1 depicts the PRISMA flowchart for the present review.

## 3. Results

Of 247 results identified in pre-screening, based on title and abstract analysis, the search results were assessed for thematic relevance (the impact of PUFAs on OLP, aphthous stomatitis, and pemphigus, including synonymous terms). A total of 162 publications were rejected as thematically non-concordant, and 85 articles were qualified for full-text analysis. Following abstract and full-text review, 79 articles were excluded (removal of studies whose results had not been published and removal of duplicates identified after article qualification across all databases). Of the 79 excluded full-text records, 66 were duplicate records identified across databases and 73 did not meet the eligibility criteria.

A total of 6 studies fulfilling all search criteria were included in the final systematic review [30,31,32,33,34,35].

### 3.1. Oral Lichen Planus

The relationship between metabolites of ω fatty acids and the clinical course of lichen planus has been described in only one clinical study, which focused strictly on tissue lipidomic profiles rather than dietary interventions. Excess ω-6 acids (linoleic acid, ALA) may, in turn, contribute to the development of inflammation through a mechanism related to the formation of oxylipins, as demonstrated by biochemical analysis of tissue specimens from patients with lichen planus. Within the lesional tissue, a profound imbalance in numerous lipid derivatives was identified, most notably 9-HODE and 13-HODE. The concentration of 9-HODE in tissue with pronounced clinical symptoms was markedly higher relative to 13-HODE compared with healthy tissue. The 9-HODE fraction, predominant in OLP foci, was shown to disrupt protective epithelial barriers and sensitize pain receptors (e.g., TRPV1), resulting in burning and tenderness of the lesions [30].

### 3.2. Recurrent Aphthous Stomatitis

The effect of ω-3 fatty acid supplementation on the clinical course of recurrent aphthous stomatitis (RAS) was analyzed in three independent clinical studies [31,32,33]. Analysis of the qualified studies suggests that ω-3 fatty acid supplementation may contribute to a significant decrease in the number of ulcerations, shortening of their duration, reduction in the level of tissue irritation, decrease in patient-reported pain levels (measured on the VAS scale) [31], and limitation of disease recurrences [32]. The first measurable therapeutic effects were observed after three months of supplementation, with stabilization occurring in the subsequent quarter of therapy [32]. The results indicate stimulation of epithelial repair processes by fatty acid supplementation, as confirmed by the absence of similar changes in the placebo-receiving control group [31]. A significant factor was an 8-month modification of the home diet’s fatty acid profile, including replacing oils high in *n*-6 fatty acids with products rich in α-linolenic acid (ALA). The implemented dietary modification results in a significant increase in plasma phospholipid ALA concentration. This intervention is associated with a significant reduction in the frequency of minor aphthae compared with the pre-study period. Importantly, this effect was observed to a comparable degree in both intervention groups (using a soy-rapeseed blend and evening primrose oil, respectively), with no statistically significant differences in clinical efficacy between these groups. The above results suggest that improving the clinical condition of oral mucosa requires optimizing overall ω-3 acid intake, regardless of the specific plant source [33].

### 3.3. Pemphigus

The evidence regarding pemphigus is strictly observational, originating from two cross-sectional studies that share the same cohort of 138 patients [34,35]. Consequently, these findings must be interpreted as a single line of cross-sectional evidence rather than two independent datasets. These interrelated clinical studies evaluated the impact of dietary quality on the course and severity of pemphigus. Analysis of dietary habits in patients with pemphigus vulgaris revealed a significant association between diet quality and clinical disease activity. High values of the Alternative Healthy Eating Index (AHEI), which characterize a diet rich in fruits, vegetables, legumes, whole-grain products, and ω-3 fatty acids (e.g., fish), correlate with reductions of 72–76% in disease severity, as measured by the PDAI index. This association was strengthened after adjusting for daily energy intake and subjects’ BMI (Body Mass Index) [34]. Interestingly, patients with a severe course of pemphigus vulgaris (>15 points on the PDAI) demonstrate statistically lower intake of fiber and vitamin C, alongside higher cholesterol intake. Regarding lipid parameters, a trend was observed suggesting that a higher ω-6/ω-3 ratio may be associated with a lower risk of severe disease course, although these results did not reach full statistical significance [35].

Table 3 depicts the main findings of the studies included in this review.

## 4. Risk of Bias Assessment

### 4.1. Randomized Studies

The reliability of the randomized clinical trials included in this work was verified using the updated Cochrane Risk of Bias 2 (RoB 2) tool. The analysis encompassed five key domains (D1–D5) and assessed the risk of bias at each stage of the research process [31,32,33].

**Figure 2:**
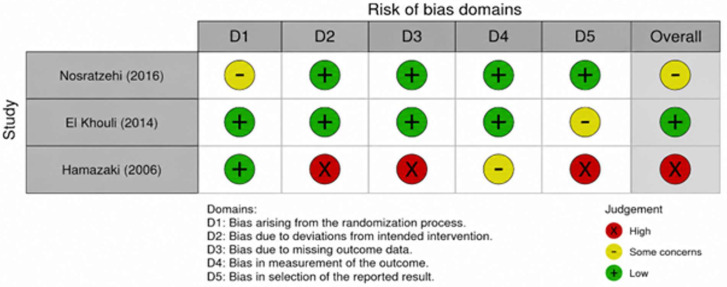
Risk of bias of the randomized clinical trials included in this review verified with the updated Cochrane Risk of Bias 2 (RoB 2) tool [31,32,33].

Figure 2 depicts the evaluation of RCTs with RoB2.

The highest methodological standard across all domains was demonstrated in the El Khouli study [32]. The authors eliminated the risk of selection and performance bias by using a computer program to generate random allocation lists and by employing a placebo identical in appearance and organoleptic properties. Such an approach guarantees complete blinding of participants and personnel, thereby ensuring the objectivity of the results. In the Nosratzehi study [31], detailed information regarding the masking of the taste and odor of the ω-3 acid capsules is lacking. This creates a risk that patients may have been able to discern the type of substance administered. This is critical in the context of pain assessment using the subjective VAS scale, as awareness of receiving active treatment may have unconsciously influenced the reported symptom intensity. In the Hamazaki study [33], areas of some concern were identified, primarily regarding outcome measurement and supervision of the conducted intervention. In this case, the intervention consisted of using appropriate cooking oils under home conditions. The principal limitation was the lack of standardization of thermal processing (variation in heating devices and cookware). This precludes maintaining a consistent smoke point, which may directly affect lipid oxidation. It is assumed that the properties of the oils consumed by patients may have differed substantially. Home conditions, in contrast to laboratory settings, impeded full control over protocol adherence—subjects could have used other oils or butters available in the household. Additionally, differences in the taste of prepared dishes may have compromised the blinding procedure, which, combined with subjects’ subjective reporting of aphthae frequency, constitutes a significant source of systematic bias, limiting the reliability of the obtained results. Across all publications, a very low patient attrition rate was observed (below 10%), ensuring high retention and allowing the final statistical analysis to include a group nearly identical to the one originally randomized. The absence of significant attrition bias is crucial for the stability of results. Had the proportion of lost patients been high (e.g., above 30%), the conclusions could have applied only to individuals with the best tolerance to the intervention, distorting the picture of therapeutic efficacy for the general population.

In summary, despite certain methodological reservations about the specificity of studies conducted under home conditions, the accumulated evidence is consistent. The Nosratzehi [31] and El Khouli [32] studies constitute the evidential foundation of the highest research integrity, whereas the Hamazaki [33] study serves as an important complement, reflecting the real-world application of dietary interventions in patients’ daily lives.

### 4.2. Non-Randomized Studies

Analogously to the RoB 2 procedure, analysis using the Newcastle–Ottawa Scale (NOS) is an integral part of assessing the risk of bias in non-randomized studies. To assess the risk of bias in study [30], the original Newcastle–Ottawa Quality Assessment Form for Case–Control Studies was utilized, whereas a modified version of the NOS adapted for cross-sectional designs was applied to studies [34,35]. Table 4 depicts the results of Newcastle-Ottawa Scale (NOS) evaluation for case-control studies.

Selection 2★/4★. The diagnosis of OLP patients was based on a tissue biopsy which represents the gold standard and provides independent medical validation of the diagnosis. For this reason, the study received 1 star in item 1.1. Due to the small sample size (*n* = 8), which is not representative of the general population an2d the lack of description of the selection criteria for the control group, no stars were awarded in items 1.2 and 1.3. Since the control group was defined as “healthy controls,” the study met the criteria for 1 star in item 1.4.

Comparability 0★/2★. The study did not report an analysis of confounding factors. The authors provided no information regarding the baseline characteristics of the groups in terms of age, sex, or other factors that could potentially influence the final outcomes.

Exposure 3★/3★. The levels of oxylipins were quantified using an objective laboratory method based on tissue biopsies. The same method was applied to both the case and control groups. Furthermore, a complete dataset was obtained from all collected tissue samples (*n* = 16), with no data attrition or losses reported during the experimental procedure.

Table 5 shows the results of Newcastle-Ottawa Scale (NOS) evaluation for cross-sectional studies.

As both reports were based on the same study population, they were evaluated jointly using a single methodological assessment, despite analyzing different variables.

Selection 3★/5★. The study met the criteria for sub-item 1b because the sample was considered reasonably representative of patients with pemphigus, despite the use of non-random sampling and a relatively small sample size (*n* = 138). However, due to the lack of information on the proportion of patients who refused to participate or withdrew, no stars were awarded for sub-items 2 and 3. In item 4, the measurement tool was assessed: the studies utilized the Food Frequency Questionnaire (FFQ) for dietary assessment and the standardized Pemphigus Disease Area Index (PDAI) scale for skin assessment. Consequently, the maximum 2 stars were awarded.

Comparability 1★/1★. The content of the articles indicates that the researchers adjusted for potential confounding factors, including demographic, anthropometric, and dietary variables. This comprehensive control for confounders justified the award of 1 star.

Outcome 3★/3★. For the Assessment of Outcome, 2 stars were awarded because disease severity was evaluated based on medical records and clinical examination, rather than subjective, self-reported patient surveys. Furthermore, the analysis utilized appropriate statistical tests for cross-sectional studies, earning an additional 1★ in the statistical test category.

In summary, all 3 papers were included in the systematic review because each achieved a score above 5★. This level of methodological quality was considered sufficient to meet the predefined inclusion criteria for the final synthesis.

### 4.3. Immortal Time Bias (ITB)

Immortal time bias occurs in time-to-event analyses when a period of follow-up is defined in such a way that the participant cannot experience the outcome of interest by definition, either intentionally or for technical reasons. This period typically occurs between cohort entry and the actual start of treatment. When this waiting time is incorrectly classified as part of the exposure period, the exposed group gains an artificial survival advantage, leading to biased estimates of treatment effect.

The study published in 2025 has shown that the presence of immortal time bias may distort results, leading to an overestimation of effect sizes in favor of the investigated intervention by up to 29%, which may consequently lead to falsely statistically significant conclusions [36].

This type of bias is primarily relevant to cohort studies and, to a lesser extent, to case–control studies evaluating time-dependent interventions. None of the included studies were at risk of immortal time bias due to their methodological design. Therefore, this bias was not considered a relevant source of bias in the present systematic review.

### 4.4. Certainty of Evidence

Since this systematic review incorporates studies evaluating three different clinical conditions, the certainty of evidence assessments using the GRADE approach have been detailed across Table 6, Table 7 and Table 8.

Due to the case–control study design, the baseline certainty of evidence is Low. Given the previously assessed risk of bias suggesting a moderate-to-high risk of systematic error, alongside the small sample size, the quality of the study was downgraded by further levels, resulting in a Very Low overall certainty of evidence rating.

Due to the randomized controlled trial (RCT) design, the baseline certainty of evidence is High. However, due to the high risk of bias in study 33 and the small sample size, the overall quality of evidence was downgraded by two levels, resulting in a Low GRADE rating.

Due to the cross-sectional study design, the baseline certainty of evidence is Low. Given the small sample size, the quality of evidence was downgraded by one level for imprecision, resulting in a Very Low GRADE rating.

## 5. Discussion

Current literature is growing an interest in dietary interventions as supportive measures in managing oral mucosal inflammatory conditions. This may stem from the fact that dietary modification carries a low risk of adverse effects, unlike the use of medications, and has already proven to have positive health effects on other systems and metabolic processes within the human body. Dietary lipid profile optimization (with an emphasis on ω-3 fatty acids) is investigated as a significant supportive measure in the management of oral lichen planus (OLP), recurrent aphthous stomatitis (RAS), and pemphigus and potentially other oral autoimmune diseases. High concentrations of 9-hydroxyoctadecadienoic acid (9-HODE, a metabolite of LA) have been associated with rise in inflammatory markers but also pain signalling. Experimental studies suggest that this molecule may contribute to the disruption of intercellular junctions and sensitization of nociceptors, potentially contributing to painful symptoms. From a mechanistic perspective, increasing dietary intake of ω-3 polyunsaturated fatty acids could theoretically influence these pathways through competition with ω-6 fatty acids for shared metabolic enzymes (Table 2), potentially shifting lipid mediator production toward specialized pro-resolving mediators (SPMs) rather than the destructive 9-HODE [30]. However, direct evidence supporting such effects in OLP is currently lacking. The study by Gouveia-Figueira et al. demonstrated altered proportions of linoleic acid-derived oxylipins in OLP lesions, suggesting that lipid mediators may be involved in disease pathogenesis and symptom generation. Because this study did not investigate dietary modification or fatty acid supplementation, it does not provide direct evidence that changing dietary fatty acid intake influences clinical outcomes in OLP. The findings should therefore be interpreted as mechanistic evidence supporting biological plausibility rather than proof of clinical effectiveness.

In the clinical study included in this review, improvement was observed following regular consumption of perilla oil, which is rich in ω-3 fatty acids. Notably, a soybean–rapeseed oil blend (containing significantly lower ω-3 levels than perilla oil) produced a similar clinical outcome [33]. This may suggest a possible “ceiling effect” (being a post hoc speculation) or saturation threshold, beyond which greater ω-3 intake does not further impact the patient’s condition. Alternatively, it may imply that clinical improvement is driven less by extreme ω-3 increases and more by the elimination of excess ω-6 (derived, e.g., from sunflower or corn oils) and the restoration of a more favorable ω-3/ω-6 ratio. Since this OLP study represents a cross-sectional mechanistic correlation rather than evidence of any effect of diet on the course of the disease, these interpretations remain speculative and require further investigation.

The results of Woelber et al., who aimed to investigate the influence of an anti-inflammatory diet on different parameters in patients with gingivitis, indicated that the evaluated diet could significantly reduce gum inflammation in a clinically relevant range, while it did not significantly modify serological inflammatory parameters and the subgingival microbiome during the study period. The experimental group in their research switched to a diet low in processed carbohydrates and animal proteins, and rich in omega-3 fatty acids, vitamin C, vitamin D, antioxidants, plant nitrates, and fiber for 4 weeks. The control group did not change their diet. Both groups suspended interdental cleaning. Compared with controls, the experimental group showed a significant reduction in gingival bleeding, a significant increase in Vitamin D levels, and significant weight loss. Meanwhile, there were no inter-group differences in the inflammatory serological parameters, the omega fatty acids, or the subgingival microbiome composition [37].

Current recommendations regarding ω-3 fatty acid requirements are inconsistent and vary by country, age group, physiological status, and sex—for instance, they differ in women during pregnancy and lactation. For instance, guidelines range from 90 mg/day (EPA + DPA + DHA) in Australia and New Zealand, to as much as 500 mg (DHA + EPA, in a 1:1 ratio) in France. The most commonly cited standard for healthy adults (7 of 18 recommendations) is a daily intake of 250 mg of EPA and DHA [38]. This highlights a strong need for further research in this area and for the establishment of unified guidelines for both healthy individuals and patients with autoimmune oral diseases. Clear and precise guidelines would enable primary care physicians to implement preventive strategies and first-line therapy based on ω-3 fatty acid supplementation and a diet characterized by a favorable ω-3/ω-6 ratio. This would allow for faster initiation of treatment, in many cases without the need for specialist consultation, while imposing only a minimal financial burden.

Alongside the well-established role of fatty acid profile, the intake of certain other well-known substances has emerged as a significant—and perhaps less intuitive—factor influencing the course of autoimmune oral diseases. In patients with severe pemphigus, a statistically lower intake of dietary fiber and vitamin C was observed, alongside elevated cholesterol intake, with these deficiencies directly correlating with the degree of clinical symptom severity [35]. Adequate intake of vitamin C and dietary fiber may help attenuate systemic inflammation by reducing oxidative stress (vitamin C as an antioxidant) and supporting gut microbiota (dietary fiber as a prebiotic). Consequently, this results in decreased autoantibody-mediated damage to keratinocytes, ultimately leading to improved clinical outcomes [39,40]. It should be noted, however, that given the cross-sectional nature of the studies, the observed correlations warrant confirmation in long-term, randomized, prospective trials.

The duration of supplementation remains a critical issue. Based on current knowledge, it remains unclear whether patients with autoimmune oral diseases should regularly and lifelong supplement ω-3 fatty acids and/or vitamin C, or whether supplementation should be reserved for specific periods, such as during disease flares or particular stages of life Table 3 [41,42].

Dietary requirements for ω-3 fatty acids can be met through both plant- and animal-derived sources. Particularly high ALA content can be found in chia seeds (*Salvia hispanica*), perilla (*Perilla frutescens*), and flaxseed (*Linum usitatissimum*), as well as in leafy green vegetables and pepper (*Capsicum annuum*). Nevertheless, the conversion of ALA to long-chain ω-3 fatty acids in humans is highly limited. For instance, the conversion rate of ALA to DHA is estimated to be only 0.01–0.05%. Therefore, relying only on plant-based sources of ω-3 fatty acids may not ensure sufficient amounts of DHA and other long-chain omega-3 fatty acids to achieve physiologically relevant levels in the body [39].

The main readily accessible sources of EPA, DPA, and DHA include marine fish oils, cold-water seafood, and marine algae oils. Minor amounts are present in meat, eggs, and dairy products, particularly when derived from pasture-raised or grass-fed animals. Nowadays marine microalgae are emerging as an excellent diet choice [40].

Given that dietary modification is a straightforward, physiologically safe, non-invasive, and generally cost-effective approach, its clinical implementation may serve as a valuable component of adjunctive therapy. Currently, our most consistent and robust evidence stems from the analysis of Recurrent Aphthous Stomatitis (RAS), as these studies achieved moderate-to-high methodological quality and demonstrated clear, reproducible clinical benefits. Consequently, these findings represent the most reliable foundation for potential therapeutic recommendations in our review. However, because the current evidence base remains limited and consists of a heterogeneous mix of observational studies and small-scale clinical trials, nutritional optimization must not be presented as a substitute for conventional pharmacological therapies. Instead, it should be viewed as a complementary strategy approach that may support systemic tissue resilience and potentially ameliorate clinical disease severity. Whether dietary interventions offer a realistic opportunity to reduce reliance on standard medications remains an open question that can be answered in future high-quality studies. Currently, further prospective and well-designed research is needed to systematically determine if targeted nutritional optimization can assist in decreasing the overall treatment burden, drug requirements, or the risk of side effects associated with long-term drug therapy. Until such definitive clinical data are available, dietary adjustments and PUFA supplementation must be viewed strictly as a complementary approach.

This review combined studies involving three distinct disease entities (oral lichen planus, recurrent aphthous stomatitis, and pemphigus), each characterized by different pathophysiological mechanisms and outcome measures. The interventions varied considerably, including omega-3 supplementation at different doses and durations, dietary replacement of cooking oils, assessment of dietary quality indices, and observational analyses of lipid profiles and fatty acid-derived metabolites. Moreover, the studies reported highly diverse outcomes, such as oxylipin concentrations (9-HODE/13-HODE ratio), ulcer size, pain intensity measured by VAS, recurrence frequency, healing time, quality of life (OHIP-14), plasma phospholipid fatty acid composition, AHEI-2010 scores, and Pemphigus Disease Area Index (PDAI) values. Many of the reported outcomes were subjective, including pain intensity (VAS), quality-of-life scores (OHIP-14), and self-reported recurrence frequency. Such measures are inherently vulnerable to reporting bias, expectation effects, and placebo responses. Although clinically important, these outcomes should be interpreted with greater caution than objective biomarkers or standardized clinician-assessed disease activity measures.

Furthermore, the included studies differed substantially in design, comprising randomized controlled trials, cross-sectional studies, and observational investigations. The inclusion of randomized, observational, cross-sectional, and mechanistic studies allowed a broader assessment of the available evidence in this emerging field. However, these study designs differ substantially in their ability to establish causality. While randomized studies provide evidence regarding intervention efficacy, observational and mechanistic studies primarily contribute information on associations and biological plausibility. Therefore, the conclusions of this review should be interpreted with appropriate caution, primarily as hypothesis-generating rather than definitive evidence of causality. Further well-designed randomized clinical trials are needed to confirm the observed relationships.

The limited number of eligible studies within each disease category and the lack of comparable quantitative outcome data precluded calculation of a meaningful pooled effect estimate. Therefore, a qualitative synthesis of the available evidence instead of meta-analysis was considered the most appropriate methodological approach. A particular limitation relates to the evidence available for oral lichen planus. Only one eligible study was identified, and this study was mechanistic in nature, evaluating tissue oxylipin profiles rather than the effects of dietary intervention. Consequently, no direct conclusions regarding the efficacy of omega-3 supplementation or dietary fatty acid modification in OLP can be drawn from the currently available evidence. Future randomized clinical trials are needed to determine whether the observed biochemical alterations translate into clinically meaningful therapeutic benefits.

Another limitation of this review is that grey literature was not searched. Consequently, unpublished studies, conference proceedings, dissertations, and other non-indexed sources were not considered. This may have increased the risk of publication bias, as studies reporting null or negative results are less likely to be published in peer-reviewed journals. Therefore, the available evidence may overrepresent positive associations between dietary factors and clinical outcomes.

The purpose of this systematic review was to investigate the impact of dietary modifications related to UFA intake on the course of three oral autoimmune conditions: OLP, RAS, and pemphigus. However, due to the heterogeneous methodologies of the reviewed studies and the lack of a universal diagnostic algorithm for UFA supplementation, the comparison of the effects of the tested interventions was severely limited. The number of studies qualified for this review is relatively low. The limitations of the studies reviewed in this study included: short follow-up periods, relatively small sample sizes, high variability in the scales used to evaluate outcomes, and heterogeneous study designs. In several studies, treatment efficacy is measured by describing the reduction in pain, evaluated using scales, mostly VAS. Pain is generally a subjective impression, not measurable with standard tools. That also may interfere with the objective evaluation of the studies’ results.

The available evidence suggests that dietary modification and optimization of unsaturated fatty acid intake may have potential as adjunctive strategies in selected oral autoimmune diseases, particularly recurrent aphthous stomatitis. However, the current evidence base remains limited by small study numbers, heterogeneous methodologies, potential publication bias, and a substantial reliance on subjective outcome measures. Therefore, these findings should be considered preliminary until confirmed by larger, well-designed randomized clinical trials employing standardized and objective outcome measures.

## Figures and Tables

**Table 1:** Summary of the most crucial epidemiologic, etiopathogenetic, and clinical features of oral disease included in the review [1,2,3,4,5,6,7,8,9].

Disease	Epidemiology	Pathomechanism	Inflammatory Markers	Clinical Presentation	Role of Mucosal Barrier	Treatment Algorithm
Oral Lichen Planus	0.5–2.0% of the general population Age of the onset: 30 to 60 years Female predilection	Cytotoxic response of Tc lymphocytes (CD8+) against keratinocytes of the basal epidermal layer Unknown antigen inducing the autoimmune mechanism	↑ IFN-γ, TNF-α, IL-2, IL-6, and IL-10 in the microenvironment of lesions	Polygonal, flat, shiny papules with red, violet coloration. Wickham’s striae visible on the surface.	Destruction of the basement membrane by lymphocytic infiltrate	Topical corticosteroids (e.g., clobetasol propionate 0.05%) Calcineurin inhibitors (tacrolimus) Retinoids
Recurrent Aphthous Stomatitis	Up to 20% of the population Onset in the second decade of life Individuals with high socioeconomic status	Multifactorial etiology: stress, mechanical trauma, genetic predisposition. Activation of T lymphocytes (mainly CD8+, Tc) and production of TNF-α	The key marker: TNF-α, ↑ IL-2, IL-12 ↓ IL-10 (in the active phase)	Painful, oval erosions or ulcers covered with fibrinous exudate with an inflammatory rim	Focal disruption of the epithelial barrier	Topical corticosteroids Protective topical agents Anesthetic agents (e.g., lidocaine) Others: chlorhexidine, colchicine, pentoxifylline
Pemphigus	0.1–0.5 per 100.000 persons annually Peak incidence between 40 and 60 years of age	Production of IgG class autoantibodies against desmoglein 3 (Dsg3) and desmoglein 1 (Dsg1), leading to acantholysis	Presence of circulating anti-Dsg3/anti-Dsg1 antibodies (detected by ELISA test) + deposits of IgG and complement C3 (in DIF examination)	Flaccid blisters, easily ruptured, leaving painful erosions Positive Nikolsky sign	Complete loss of epidermal/mucosal barrier integrity is observed as a result of acantholysis, leading to massive fluid loss and risk of sepsis	Systemic corticosteroids (e.g., prednisone in high doses) Immunosuppressives (azathioprine, mycophenolate mofetil, rituximab)

**Table 2:** Biological and immunological effects of unsaturated fatty acids [19,24,25,26,27,28,29].

	Process Description	Proven Biological and Immunological Effect
Immuno- modulation	Blocking of NF-κB pathways with simultaneous activation of PPAR-γ receptors Suppression of IL-2 secretion by *n*-3 series acids	↓IL-1, IL-6, and TNF-α concentrations in serum and tissues; TCR blockade, ↓IL-2 levels, ↓T lymphocyte proliferation (especially Th1) Anti-inflammatory action and inhibition of autoaggression processes
Resolution of Inflammation	Synthesis of specialized pro-resolving mediators (SPMs): resolvins, protectins, and maresins from EPA and DHA acids	These molecules accelerate the resolution of inflammation and stimulate tissue repair by inhibiting the production of cytokines TNF-α, IL-1β, and IL-6 in macrophages and monocytes
Oxidative Stress	Limitation of NADPH oxidase activity and production of reactive oxygen species (ROS)	Protection of cellular structures from damage caused by free radicals; reduction in DNA and membrane lipid damage

**Table 3:** Detailed summary of study data and findings [30,31,32,33,34,35].

Disease	Country	Population (Sample Size, Age, Sex, and Others)	Intervention (Dose, Treatment Scheme)	Evaluation Criteria	Time of Observation	Results
Oral Lichen Planus	[30] Sweden (Umeå University)	16 participants: 8 OLP patients (4 females, 4 males; age range 43–79, median 61) and 8 healthy volunteers (6 females, 2 males; age range 43–67, median 50)	4 mm punch biopsies from buccal mucosa; ex vivo analysis of linoleic acid derivatives using UPLC-MS/MS	Proportions of LA-derived oxylipins (9-HODE, 13-HODE, etc.); log-ratio transformations; MANOVA statistical evaluation	Single point in time (cross-sectional biopsy study)	The ratio of 9-HODE to 13-HODE was 4.1-fold higher in OLP biopsies compared to control biopsies (*p* = 0.026)
Recurrent Aphthous Stomatitis	[31] Iran (Zahedan University of Medical Sciences)	50 patients (25 omega-3 group, 25 placebo); ages 16–54; mean age ~31–37 years	1000 mg omega-3 capsules taken 3x daily for 6 months	Pain (VAS 0–10), ulcer size (mm), duration, and recurrence rate	6 months (October 2013–December 2015)	Significant reduction in pain (4.96 to 3.04), ulcer size (2.30 to 1.48 mm), and number of ulcers/recurrence by months 5–6 (*p* < 0.05)
	[32] Egypt (October 6 University, Giza)	50 participants (25 omega-3, 25 placebo); age ≥ 13; history of minor RAS for ≥ 1 year	3000 mg omega-3 daily (1000 mg capsule 3x/day) for 6 months; each capsule contained 300 mg EPA and 200 mg DHA	Monthly number of ulcers, average pain (VAS), duration of episodes, and quality of life (OHIP-14)	6 months (April 2012–September 2013)	Significant reduction in ulcers, pain, and duration starting from month 3. OHIP-14 scores significantly improved by month 6 (*p* < 0.01)
	[33] Japan (Toyama prefecture and Tokyo metropolitan area)	30 participants (8 men, 22 women); ages 21–70; minor RAS frequency ≥ once a month	Dietary change to perilla oil (rich in ALA) or soybean-rapeseed oil blend as the sole cooking oil	Prevalence of RAS, healing time, and fatty acid analysis of total plasma phospholipid fraction	12 months (4-month run-in phase followed by an 8-month experimental phase)	RAS prevalence decreased significantly in both groups; Perilla oil was not superior to Soybean oil
Pemphigus	[34] Iran (Tehran University of Medical Sciences)	138 participants; 18–65 years of age; Iranian population; 1.6:1 female-to-male ratio	High adherence to the Alternative Healthy Eating Index 2010 (AHEI-2010); dietary intake assessed via 168-item semi-quantitative food frequency questionnaire (SFFQ)	Pemphigus Disease Area Index (PDAI) for disease severity (mild ≤ 15, severe > 15); AHEI-2010 score (9–90 points) for diet quality	Cross-sectional study; conducted from December 2021 to May 2022	Highest AHEI adherence (Q4) associated with 82% reduced odds of severe PV (OR: 0.18; 95% CI: 0.04–0.82) compared to the lowest quartile
	[35] Iran (Tehran University of Medical Sciences)	138 PV cases (108 mild, PDAI ≤ 15; 30 severe, PDAI > 15), ages 18–65	Dietary lipid indices assessment (ω-6/ω-3 ratio and PUFA/SFA ratio) via 168-item FFQ	Pemphigus Disease Area Index (PDAI); lipid index calculations based on dietary fat intake	Cross-sectional study (December 2021–May 2022)	Replacing inflammatory ω-6 cooking oils with ω-3 alternatives reduced the prevalence of oral lesions, although no significant association found between ω-6/ω-3 (Ptrend = 0.47) or PUFA/SFA (Ptrend = 0.88) and PV severity

**Table 4:** The results of the Newcastle–Ottawa Scale (NOS) evaluation of studies with case–control designs.

Study	Selection	Comparability	Exposure	Overall	Comment
Gouveia-Figueira, S (2019) [30]	2★	0★	3★	5★	Low to moderate quality

**Table 5:** The results of the Newcastle–Ottawa Scale (NOS) evaluation of studies with cross-sectional designs.

Study	Selection	Comparability	Outcome	Overall	Comment
Fallah, M (2023) [34] Fallah, M (2025) [35]	3★	1★	3★	7★	Low to moderate quality

**Table 6:** GRADE Evidence Profile for Oral Lichen Planus.

Study	Study Design	Risk of Bias	Imprecision	GRADE
Gouveia-Figueira, S 2019 [30]	case–control	Serious (Low to moderate quality)	Very serious (*n* = 16)	Very Low (⊕ ⊖ ⊖ ⊖)

**Table 7:** GRADE Evidence Profile for Recurrent Aphthous Stomatitis.

Study	Study Design	Risk of Bias	Imprecision	GRADE
Nosratzehi (2016) [31] El Khouli (2014) [32] Hamazaki (2006) [33]	RCT	Serious	Serious (*n* = 130)	Low (⊕ ⊕ ⊖ ⊖)

**Table 8:** GRADE Evidence Profile for Pempfigus.

Study	Study Design	Risk of Bias	Imprecision	GRADE
Fallah, M (2023) [34] Fallah, M (2025) [35]	Cross-sectional study	Not serious (moderate to high quality)	Serious (*n* = 138)	Very Low (⊕ ⊖ ⊖ ⊖)

## Data Availability

Data sharing is not applicable to this article as no new datasets were generated; the study relies entirely on previously published literature.

## Supplementary Materials

The following supporting information can be downloaded at: https://www.mdpi.com/article/10.3390/jcm15145542/s1, Table S1: PRISMA 2020 checklist [43].

## Appendix A. Search Strategy

PUMS Library, that includes: MEDLINE, MEDLINE Ultimate, Complementary Index, Academic Research Ultimate, Springer Nature Journals, Dentistry & Oral Sciences Source, Directory of Open Access Journals (DOAJ), Supplemental Index, OVID Journals, Health Source: Nursing/Academic Edition (EBSCO), EBSCOhost, Business Source Ultimate, MasterFILE Premier, Health Source—Consumer Edition, Research Starters, Books at JSTOR, CINAHL Ultimate, ScienceDirect, Scopus, Web of Science, Wiley Online Library, DynaMed, Oxford University Press, Cambridge University Press, AccessMedicine, ClinicalKey, Cochrane Library, Embase, LWW Health Library, Nature Journals, ProQuest Central, PsycINFO, Reaxys, UpToDate, Bibliography of PUMS Staff Publications, PUMS Repository, Legimi, Ibuk Libra

*((“unsaturated fatty acid*” OR “n-3 fatty acid*” OR “omega-3 fatty acid*” OR “omega 3” OR PUFA OR “polyunsaturated fatty acid*”) AND (“oral lichen planus” OR “lichen planus” OR “pemphigus vulgaris” OR “pemphigus”))*

Embase/Elsevier:

*(‘unsaturated fatty acid’/exp OR ‘unsaturated fatty acid’:ti,ab OR ‘polyunsaturated fatty acid’/exp OR ‘pufa’:ti,ab OR ‘omega 3 fatty acid’/exp OR ‘omega-3’:ti,ab OR ‘n-3 fatty acid’:ti,ab) AND (‘oral lichen planus’/exp OR ‘pemphigus’/exp OR ‘lichen planus’:ti,ab OR ‘oral lichen planus’:ti,ab OR ‘stomatitis’:ti,ab OR ‘pemphigus’:ti,ab)*

PubMed/MEDLINE

*(“Diet”[Mesh] OR “Dietary Fats, Unsaturated”[Mesh] OR “Fatty Acids, Unsaturated”[Mesh] OR “Diet Modification” OR “Polyunsaturated Fatty Acids”) AND (“Lichen Planus, Oral”[Mesh] OR “Stomatitis, Aphthous”[Mesh] OR “Pemphigus”[Mesh] OR “Oral Autoimmune Diseases”)*

Scopus:

*TITLE-ABS-KEY(“unsaturated fatty acid” OR “polyunsaturated fatty acid” OR “pufa” OR “omega 3 fatty acid” OR “omega-3” OR “n-3 fatty acid” OR “diet modification”) AND TITLE-ABS-KEY(“oral lichen planus” OR “pemphigus” OR “stomatitis” OR “oral autoimmune diseases”)*

Web of Science:

*TS=(“unsaturated fatty acid” OR “polyunsaturated fatty acid” OR “pufa” OR “omega 3 fatty acid” OR “omega-3” OR “n-3 fatty acid” OR “diet modification”) AND TS=(“oral lichen planus” OR “pemphigus” OR “stomatitis” OR “oral autoimmune diseases”)*

(2853 results, distributed as follows):

MEDLINE (102), MEDLINE Ultimate (486), Scopus (463), Complementary Index (1005), Academic Research Ultimate (387), Springer Nature Journals (157), Dentistry & Oral Sciences Source (89), Embase (88), Web of Science (35), Directory of Open Access Journals (DOAJ) (4), Supplemental Index (1), Health Source: Nursing/Academic Edition (EBSCO) (10), EBSCOhost (14), Business Source Ultimate (6), MasterFILE Premier (2), Health Source—Consumer Edition (1), Research Starters (1), Books at JSTOR (1), rest (0)

*Pre-Screening*

Following the application of automated filters: identification of original studies for qualitative analysis (Adaptive Clinical Trial, Case Reports, Clinical Study, Clinical Trial, Clinical Trial Protocol, Clinical Trial Phase I–IV, Comparative Study, Controlled Clinical Trial, Evaluation Study, Multicenter Study, Observational Study, Pragmatic Clinical Trial, Randomized Controlled Trial), exclusion of studies not meeting the criteria (Reviews, Systematic Reviews, Scoping Review, Meta-Analysis, Evidence Synthesis, Clinical Trial Protocol, Conference Proceedings, Retracted Publication, Datasets, veterinary studies), and retention of texts written in the English language only.

PUMS Library:

*AND (“clinical trial” OR “clinical study” OR “case report” OR “cohort study” OR “case-control study” OR “cross-sectional study” OR “randomized controlled trial”) NOT (“review” OR “systematic review” OR “meta-analysis” OR “editorial” OR “letter” OR “conference proceeding” OR “protocol” OR “note”)*

Embase/Elsevier:

*AND (‘clinical article’/de OR ‘clinical study’/de OR ‘case control study’/de OR ‘case report’/de OR ‘cohort analysis’/de OR ‘controlled clinical trial’/de OR ‘cross sectional study’/de OR ‘observational study’/de OR ‘randomized controlled trial’/de) NOT (‘review’/de OR ‘systematic review’/de OR ‘meta analysis’/de OR ‘editorial’/de OR ‘letter’/de OR ‘conference abstract’/de OR ‘note’/de OR ‘protocol’/de)*

PubMed/MEDLINE

*AND (“Clinical Study”[PT] OR “Case Reports”[PT] OR “Clinical Trial”[PT] OR “Comparative Study”[PT] OR “Controlled Clinical Trial”[PT] OR “Observational Study”[PT] OR “Randomized Controlled Trial”[PT] OR “Evaluation Study”[PT] OR “Multicenter Study”[PT]) NOT (“Review”[PT] OR “Systematic Review”[PT] OR “Meta-Analysis”[PT] OR “Editorial”[PT] OR “Letter”[PT] OR “Congresses”[PT] OR “Clinical Trial Protocol”[PT])*

Scopus:

*AND (TITLE-ABS-KEY(“clinical study” OR “case report” OR “case control study” OR “cohort study” OR “controlled clinical trial” OR “cross sectional study” OR “observational study” OR “randomized controlled trial”)) AND NOT (TITLE-ABS-KEY(“review” OR “systematic review” OR “meta-analysis” OR “editorial” OR “letter” OR “conference abstract” OR “note” OR “protocol”))*

Web of Science:

*AND (TS=(“clinical study” OR “case report” OR “case control study” OR “cohort study” OR “controlled clinical trial” OR “cross sectional study” OR “observational study” OR “randomized controlled trial”)) NOT (TS=(“review” OR “systematic review” OR “meta-analysis” OR “editorial” OR “letter” OR “conference proceeding” OR “protocol”))*

(247 results, distributed as follows):

MEDLINE (27), MEDLINE Ultimate (24), Complementary Index (92), Embase (33), Academic Research Ultimate (27), Scopus (21), Web of Science (20), Springer Nature Journals (3), Dentistry & Oral Sciences Source (5), Directory of Open Access Journals (DOAJ) (2), Health Source: Nursing/Academic Edition (EBSCO) (2), Business Source Ultimate (3), MasterFILE Premier (1), Health Source—Consumer Edition (1), rest (0)

*Screening*

Based on the analysis of titles and abstracts, the search results were assessed for thematic relevance (the impact of PUFAs on OLP, aphthous stomatitis, and pemphigus, including synonymous terms). A total of 162 publications were rejected as thematically non-concordant, and 85 articles were qualified for full-text analysis.

*Eligibility:*

Following full-text review, 79 articles were excluded (removal of studies whose results had not been published and removal of duplicates identified after article qualification across all databases).

*Included:*

A total of 6 studies fulfilling all search criteria were included in the final systematic review [30,31,32,33,34,35].

Table A1 presents the summary of the search results.

**Table A1:** Summary of the search results.

Base	Results	Pre-Screening	Screening	Eligibility
EMBASE	88	33	12	3 [32,33,34]
PUM Library	2194	148	56	4 [30,31,32,35]
PUBMED	73	25	11	3 [31,32,33]
WEB OF SCIENCE	35	20	4	1 [30]
SCOPUS	463	21	2	2 [34,35]

** PUM library includes: MEDLINE, MEDLINE Ultimate, Complementary Index, Academic Research Ultimate, Springer Nature Journals, Dentistry & Oral Sciences Source, Directory of Open Access Journals (DOAJ), Supplemental Index, OVID Journals, Health Source: Nursing/Academic Edition (EBSCO), EBSCOhost, Business Source Ultimate, MasterFILE Premier, Health Source—Consumer Edition, Research Starters, Books at JSTOR, CINAHL Ultimate, ScienceDirect, Scopus, Web of Science, Wiley Online Library, DynaMed, Oxford University Press, Cambridge University Press, AccessMedicine, ClinicalKey, Cochrane Library, Embase, LWW Health Library, Nature Journals, ProQuest Central, PsycINFO, Reaxys, UpToDate, Bibliography of PUMS Staff Publications, PUMS Repository, Legimi, Ibuk Libra*
